# Rapid gut microbiome changes in a world‐class ultramarathon runner

**DOI:** 10.14814/phy2.14313

**Published:** 2019-12-23

**Authors:** Gregory J. Grosicki, Ryan P. Durk, James R. Bagley

**Affiliations:** ^1^ Biodynamics and Human Performance Center Georgia Southern University Savannah Georgia; ^2^ San Francisco State University San Francisco California

**Keywords:** endurance exercise, gut microbiota, ultramarathon, *Veillonella*

## Abstract

The human gut microbiome is a dynamic ecosystem with prolific health connotations. Physical activity is emerging as a potent regulator of human microbiome composition. This study examined changes in the gut microbiome of a world‐class ultramarathon runner before and after competing in the Western States Endurance Run (WSER), a 163 km mountain footrace. Anthropometrics and body composition were assessed and the ultramarathoner's submaximal and maximal performance profiles were evaluated. Gut microbiome analyses were performed at four time‐points: 21 weeks and 2 weeks before and 2 hours and 10 days after WSER. Aerobic power (VO_2_max) was 4.24 L/min (66.7 ml kg^−1^ min^−1^), and running economy (51.1 ml kg^−1^ min^−1^ at 268 m/min) and lactate threshold (~83% VO_2_max) values were comparable to that of highly trained distance runners. Two hours post‐race, considerable changes in the ultrarunners’ gut microbiome were observed. Alpha diversity (Shannon Diversity Index) increased from 2.73 to 2.80 and phylum‐level bacterial composition (Firmicutes/Bacteroidetes ratio) rose from 4.4 to 14.2. Underlying these macro‐level microbial alterations were demonstrable increases in select bacterial genera such as *Veillonella* (+14,229%) and *Streptococcus* (+438%) concomitant with reductions in *Alloprevotella* (−79%) and *Subdolingranulum* (−50%). To our knowledge, this case study shows the most rapid and pronounced shifts in human gut microbiome composition after acute exercise in the human literature. These findings provide yet another example of how exercise can be a powerful modulator of human health.

## Graphical abstract

This study highlights the extent to which physical activity can rapidly transform the human gut microbiome and provides compelling impetus for continued exploration of exercise as a preventative and/or therapeutic countermeasure for the promotion of gut health.

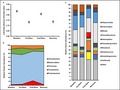

## 1 INTRODUCTION

In 2007 the National Institutes of Health launched the Human Microbiome Project (HMP), an interdisciplinary research initiative seeking to characterize the contribution of human gut microbiota to health and disease (Turnbaugh et al., 2007). Subsequent findings have demonstrated compelling relationships between human gut microbiome composition and many leading causes of death worldwide including cardiovascular disease (Wang et al., 2011), diabetes (Larsen et al., 2010), and cancer (Ahn et al., 2013). Although the gut microbiome is suggested to exhibit exceptional plasticity (Gomez et al., 2019), a detailed understanding of the factors determining human microbiome assembly is lacking (Relman, 2015).

Recently, our group (Durk et al., 2018) and others (Allen et al., 2018; Keohane et al., 2019; Scheiman et al., 2019) have shown a link between physical activity and human microbiome composition, aiding in the delineation of a signature microbiome response to exercise training. Increases in bacterial diversity and a proliferation of taxa responsible for the production of short chain fatty acids, such as butyrate, are among the most pervasively observed microbial alterations with exercise (Mailing, Allen, Buford, Fields, & Woods, 2019). While these changes are generally regarded as beneficial to the host, a comprehensive understanding of exercise training‐induced microbial modifications and their systemic physiological implications remains to be delineated. Moreover, even less is known regarding the human gut microbiome response to an acute exercise bout. Recently, we had the unique opportunity to track the gut microbiome of a world‐class ultramarathon runner who finished in the top‐10 at the Western States Endurance Run, a 163 km mountain footrace featuring ~5,486 m climbing and ~7,010 m of descent. Substantially reduced splanchnic blood flow over the course of the ~16‐hr event in concert with tremendous energetic demands (13,000–16,000 kcal) (Cuddy, Slivka, Hailes, & Dumke, 2009) provides a unique lens through which to study the plasticity of our microbial inhabitants under extreme conditions.

## 2 METHODS

### 2.1 Participant

The participant was a 32 yr old male world‐class ultramarathon runner, studied over the course of a 21‐week period during the 2019 race season. He began running competitively in high school, and in college he was a two‐time NCAA Division II Cross‐Country All‐American. He since raced in the Olympic marathon trials and is a two‐time champion at the Javelina Jundred.

### 2.2 Approval and screening

**Figure 1:**
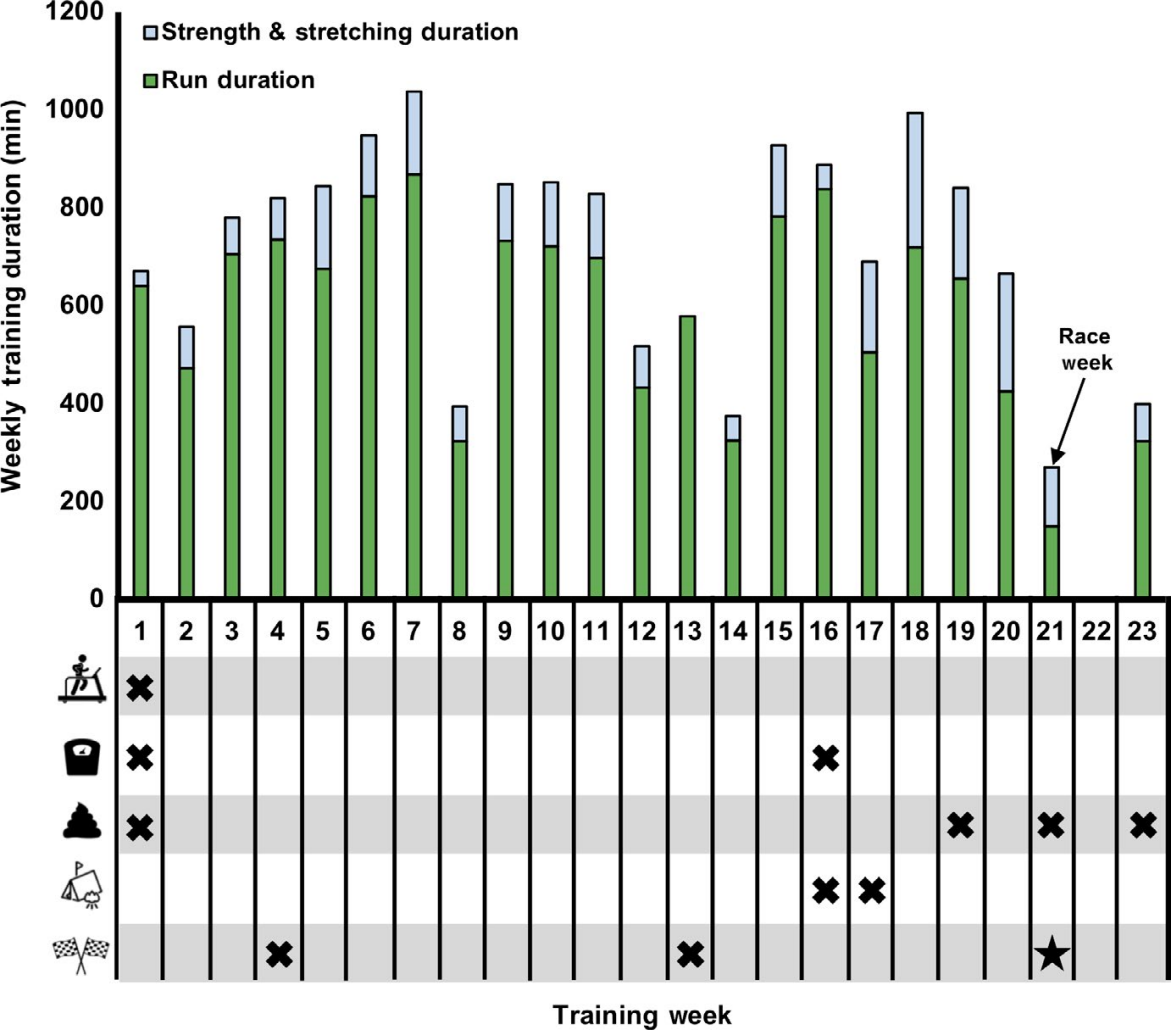
Visual representation of study design. Anthropometric and physiological measurements including cardiorespiratory fitness (treadmill) were first taken 21 weeks prior to Western States Endurance Run (WSER; 163 km mountain footrace from Squaw Valley, CA to Auburn, CA) and are reported in the text as Baseline results. Body composition (scale) and gut microbiome composition (fecal symbol) were intermittently evaluated throughout the observational period. Relevant events prior to the race such as a training camp (tent) and shorter/preparatory races (race flags) have been highlighted for temporal interpretation

This project was approved by the Institutional Review Board at Georgia Southern University. On the day of the first study visit, project objectives and risks and procedures were verbally explained to the participant, after which the participant provided written informed consent to participate. A visual representation of the study methodology is provided in Figure 1.

### 2.3 Training quantification

Daily training logs recorded via a Suunto Ambit3 Peak GPS watch provided by the ultramarathoner were utilized to quantify weekly and cumulative training duration, mileage, and pace.

### 2.4 Body composition and bone mineral content

Height and body mass were measured on a wall‐mounted stadiometer and calibrated digital scale, respectively. Body composition was assessed at 21 and 5 weeks prior to the Western States Endurance Run (WSER) (Figure 1) using dual‐energy X‐ray absorptiometry (DEXA) (Lunar iDXA, GE Healthcare). The DEXA machine was outfitted with enCORE version 16 and the machine was calibrated immediately prior to each scan per manufacturer instructions (laboratory coefficient of variation < 0.07%). Scans were obtained after an overnight fast (>10 hr) and after voiding of the bladder using “standard thickness” mode based on the subject's size characteristics.

### 2.5 Submaximal and maximal oxygen consumption (VO_2_max) exercise testing

Submaximal exercise testing was performed on a slat belt treadmill (4Front, Woodway) 30 min after ingesting a 150 kcal (23 g carbohydrate, 6 g fat, 1 g protein) solid snack (GU Energy Labs). Testing was performed in a temperature‐controlled environment (22°C, 63% relative humidity and 766.1 mmHg) using a protocol from McMiken and Daniels (1976) to facilitate running economy comparisons to a well‐characterized cohort of high‐caliber distance runners (Conley & Krahenbuhl, 1980; Costill, Thomason, & Roberts, 1973). Testing stages were 6 min in duration and expired gas was monitored throughout the test via indirect calorimetry (TrueOne2400, Parvo Medics) while heart rate was monitored via wireless telemetry (Polar H10, Polar USA). At the completion of each testing stage, blood lactate was evaluated via finger‐stick (Lactate Scout+, EKF Diagnostics). Submaximal testing was performed at a 0% grade using incremental steps in velocity (80, 134, 215, 268, 295 and 311 m/min) and proceeded until blood lactate exceeded 4 mmol/l. Gas values (e.g., O_2_ consumption, respiratory exchange ratio, etc.) were quantified using the average of two 30 s values during the last minute of each testing stage and rating of perceived exertion (RPE, Borg scale) (Borg, 1970) was assessed in the last 15 s of each stage.

Based on a recent report of performance testing in a world‐class cyclist (Bell, Furber, Someren, Anton‐Solanas, & Swart, 2017), a 15 min rest period was provided between submaximal and maximal (VO_2_max) treadmill exercise testing. The VO_2_max test was administered in 2‐min stages at 0% grade, beginning at 80 m/min and working up to the velocity at lactate threshold (295 m/min). After 2 min at this velocity, the grade was increased to 4% and then another 2% for every 2 min thereafter until volitional exhaustion. Oxygen consumption, heart rate and RPE were monitored using the same equipment and testing procedures as described above. Attainment of VO_2_max was verified by (a) achievement of greater than or equal to 90% age‐predicted maximal heart rate, (b) a respiratory exchange ratio (RER)> 1.1, and (c) a final RPE > 17.

### 2.6 Gut microbiome analyses

Gut microbiome analyses were performed at four time‐points (21 and 2 weeks pre‐WSER, 2 hours and 10 days post‐WSER), as shown in Figure 1. Stool samples were self‐collected by the ultramarathoner using a commercially available kit (Ubiome Explorer) in accordance with the specifications laid out by the NIH Human Microbiome Project (McInnes, 2010). All samples, besides the 2 hr post sample, were taken at approximately the same time of day before eating or exercising (0800). Following a bowel movement, a sterile swab was used to transfer a small amount of fecal matter into a vial containing a lysis and stabilization buffer that preserves the genetic material for transport at ambient temperatures. Samples were sent to Ubiome laboratories (Ubiome) (Bik et al., 2018) and lysed by bead‐beating prior to DNA extraction in a class 1,000 clean room using a guanidine thiocyanate silica column‐based purification method via a liquid‐handling robot. PCR amplification of the 16S rRNA genes was performed with primers containing universal primers amplifying the V4 variables region (515F: GTGCCAGCMGCCGCGGTAA and 806R: GGACTACHVGGGTWTCTAAT) (Caporaso et al., 2011). In addition, the primers contained Illumina tags and barcodes. Samples were barcoded with a unique combination of forward and reverse indexes allowing for simultaneous processing of multiple samples. PCR products were pooled, column‐purified, and size selected through microfluidic DNA fractionation (Minalla & Dubrow, 2001). Consolidated libraries were quantified using quantitative real‐time PCR using the Kapa iCycler qPCR kit (Bio‐Rad) on a BioRad Myio before loading into the sequencer. Sequencing was performed in a pair‐end modality on a NextSeq 500 platform (Illumina) rendering 2 × 150 bp pair‐end sequences. These DNA sequencing techniques were then used to generate data outputs (.csv file) that provided a comprehensive bacterial taxonomic profile. Shannon diversity index (i.e., alpha diversity) was computed using PAST: Paleontological statistics software package for education and data analysis (version 3.25) (Hammer et al., 2001).

## 3 RESULTS

### 3.1 Training quantification

For the 6 weeks prior to the WSER‐specific training block, the athlete was running ~115 km per week over mostly flat ground with little strength training. Over the course of the 21‐week training block, the participant increased volume slightly (~124 km at an average pace of 201 m/min) and supplemented with an additional 120 min per week of strength and stretching exercises, accumulating 15,331 total minutes of training time. The largest volume of training consisted of an eight‐day training camp in northern California 4–5 weeks prior to WSER (Figure 1), during which the ultramarathon runner accumulated 1,037 total minutes of training time (987 min running) and covered 177 km with 6,016 m elevation gain. To prepare for WSER, the athlete competed in a 50 k trail race at week 4, much of which was on the WSER trail, as well as a 100 k sponsor‐endorsed road race. During the 10‐day post‐race observation period, the athlete refrained from any structured exercise for seven days before beginning light jogging (~5:30/km) for 30–60 min duration.

### 3.2 Body composition and bone mineral content

Baseline body mass and height were 64.0 kg and 170 cm, respectively, and body fat mass was 14.8%. Regional distribution of baseline body fat mass was 17.2%, 12.8% and 13.7% for the arms, legs and trunk, respectively. Bone mineral content was 2.6 kg and total body bone mineral density was 1.074 g/cm^2^ (NHANES/Lunar T‐score = −1.3) (WHO Study Group, 1994). After 15 weeks of WSER‐specific training, body mass increased slightly (+0.5 kg) and total body fat was reduced to 14.2% due to compositional shifts in the arms and legs (16.5% and 12.7%, respectively). Although bone mineral content was identical between visits, bone mineral density improved to 1.086 g/cm^2^ (T‐score = −1.1), with the most notable changes occurring in the spine and pelvis (Table 1).

**Table 1:** Regional bone mineral density (g/cm^2^) in a world‐class ultramarathon runner preparing for the Western States 100‐mile Endurance Race

	Baseline	Pre‐race	%Δ
Head	1.931	1.910	−1.088
Arms	0.647	0.653	+0.927
Legs	1.264	1.273	+0.712
Trunk	0.890	0.907	+1.910
Ribs	0.739	0.742	+0.406
Spine	0.970	0.992	+2.268
Pelvis	0.984	1.014	+3.048
Total	1.074	1.086	+1.117

### 3.3 Submaximal and maximal oxygen consumption (VO_2_max) exercise testing

Submaximal testing lasted 24 min, concluding after completion of a 6‐min stage at 311 m/min (~5:10 min/mile) where blood lactate reached 6.1 mmol/l and relative oxygen consumption (VO_2_) was 61.8 ml kg^−1^ min^−1^. At velocities of 268 and 295 m/min, VO_2_ was 51.1 and 55.3 ml kg^−1^ min^−1^, running economy values comparable or even superior to those reported by Costill, Thomason and Roberts (51.7 and 59.0 ml kg^−1^ min^−1^ at corresponding velocities) in highly trained distance runners (Costill et al., 1973).

Maximal exercise testing was terminated upon volitional exhaustion (RPE = 20) at 295 m/min at an 8% grade. Maximal oxygen consumption (VO_2_max) was 4.24 L/min (66.7 ml kg^−1^ min^−1^) and maximal heart rate and RER values were 186 bpm and 1.19, respectively. Maximal ventilation was 130.7 L/min. Based on these maximal values, lactate threshold was estimated to occur at ~83% VO_2_max (RER = 0.89).

### 3.4 Gut microbiome analyses

**Figure 2:**
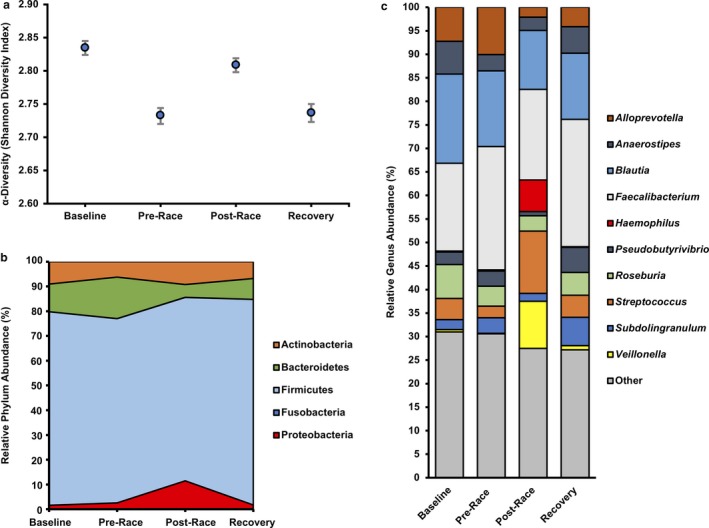
Tracking the microbiome of a world‐class ultramarathon runner. (a) Alpha diversity, represented as Shannon Diversity Index (H), of the gut microbiome in a world‐class ultramarathon runner measured at Baseline (21 week pre‐Western States Endurance Run [WSER; 163 km mountain footrace]), Pre‐Race (2 week pre‐WSER), Post‐Race (2 hr post‐WSER), and Recovery (10 days post‐WSER) measured via 16S rRNA sequencing. (b) Relative phylum‐level gut microbiome composition changes at the same time‐points listed above. (c) Relative abundance of bacteria genera over the course of the investigation. Genera comprising ≥5% of total fractional abundance for at least one time‐point are individually represented while genera of lower abundance were condensed (Other)

Microbial diversity (Shannon Diversity Index) oscillated throughout the investigation, decreasing following 19‐week of highly specific race preparation but then increasing post‐event (Figure 2a). Firmicutes/Bacteroidetes ratio, a macro‐level indicator of microbial composition, was relatively stable pre‐WSER (~5:1), but nearly tripled 2 hr post‐race due to a 69% reduction in Bacteroidetes relative abundance (Figure 2b). Meanwhile, the relative proportion of Proteobacteria increased by more than fivefold 2 hr post‐race, largely owing to a 29‐fold increase in *Haemophilus* (Figure 2c). Other notable changes in gut microbiome composition post‐WSER included the proliferation of *Veillonella* (+14,229%) and *Streptococcus* (+438%) genera concomitant with reductions in *Alloprevotella* (−79%) and *Subdolingranulum* (−50%).

## 4 DISCUSSION

Manipulating the human microbial ecosystem has prolific health implications and emerging therapeutic potential (Khanna & Tosh, 2014). To our knowledge, this case study shows the most rapid and pronounced gut microbiome changes after acute exercise in the human literature. These extraordinary microbial dynamics highlight the importance of physical activity in determining human microbiome assembly and emphasize yet another way in which human movement can be one of the most powerful modulators of human health.

Taxonomic richness (i.e., alpha diversity) is often considered a key indicator of gut microbiome health that is generally thought to increase with exercise training (Estaki et al., 2016), as was recently observed throughout an ultra‐endurance rowing race (Keohane et al., 2019). Paradoxically, in this study alpha diversity, depicted as Shannon Diversity Index, decreased following 19 weeks of highly specific race preparation (2.83–2.73). This observation may be attributed to the proliferation of select advantageous bacterial taxa, such as those involved in butyrate production (e.g., *Faecalibacterium*, +40% baseline to pre‐race), concomitant with the decline of less relevant microorganisms. In contrast, unpublished observations from our laboratory in a cohort of 28 young recreationally active individuals (~30 years) demonstrated alpha diversity changes of plus or minus 0.05 over a 3‐week time period. Post‐race, alpha diversity increased by a similar extent (+0.07) nearly reaching baseline levels (2.80). Of the post‐race changes in relative genus abundance, a likely beneficial 143‐fold increase in *Veillonella* (~10% relative abundance post‐WSER) was the most pronounced. Indeed, Scheiman et al. (2019) recently proposed an ergogenic role for *Veillonella* involving lactate recycling after observing a similar, albeit less profound, elevation (~3% relative abundance) in *Veillonella* of runners 1–5 days after the Boston Marathon.

While an increase in *Veillonella* abundance was likely a highly favorable adaptation to the 163 km race, other microbial dynamics such as post‐race insurgences of *Haemophilus*, a bacterial genus composed of many significant pathogenic species (e.g., *H. Influenzae)* and *Streptococcus* (genus‐level taxon of Group A *Streptococcus pyogenes*) were also observed. It may be speculated that intestinal proliferation of pathogenic bacterial species plays a role in the increased incidence of infectious episodes observed in endurance athletes following prolonged endurance exercise (Nieman, Johanssen, Lee, & Arabatzis, 1990). Moreover, reductions in butyrate‐producing bacteria (e.g., *Subdolingranulum)* associated with mucosal integrity likely exacerbate this state of hypervulnerability to infection as well as contributing to elevated levels of circulating inflammation (Gill et al., 2015; Grosicki, Fielding, & Lustgarten, 2018) and gastrointestinal distress (Jeukendrup et al., 2000). Surprisingly however, the athlete did not report any significant gastrointestinal complaints during or following the event. Interpreted together, genus‐level shifts in gut microbiome composition post‐WSER highlight the complexity of interpreting microbiome data and reinforce the importance of avoiding oversimplifying macro‐level observations (e.g., F/B ratio and/or alpha diversity) of microbial community structure (Shade, 2017).

In conclusion, these data add to a growing body of literature demonstrating the potency of acute exercise in shaping human microbiome composition. Though other factors (e.g., diet, travel, etc.) may have influenced our findings, no purposeful changes in diet or feelings of malaise were reported by the ultramarathon runner over the course of the data collection period. Nonetheless, more research involving carefully structured training studies in both healthy and clinical populations and interdisciplinary research teams is needed to fully understand the complex interaction between physical activity and the gut microbiome.

## CONFLICT OF INTEREST

The authors have no conflict to disclose.

## AUTHOR CONTRIBUTIONS

GJG and JRB conceived and designed the research. GJG, RPD, and RJB performed the experiments, analyzed data, interpreted results of experiments, and prepared the figures. GJG drafted the manuscript. GJG, RPD, and JRB edited, revised, and approved the final version of the manuscript.
